# Changes in Lipid Indices in HIV+ Cases on HAART

**DOI:** 10.1155/2019/2870647

**Published:** 2019-02-05

**Authors:** Shujing Ji, Yufan Xu, Dating Han, Xiuming Peng, Xiangyun Lu, Norbert H. Brockmeyer, Nanping Wu

**Affiliations:** ^1^State Key Laboratory for Diagnosis and Treatment of Infectious Diseases, The First Affiliated Hospital, School of Medicine, Zhejiang University, Hangzhou 310003, China; ^2^Collaborative Innovation Center for Diagnosis and Treatment of Infectious Diseases, Hangzhou 310003, China; ^3^Department of Dermatology and Allergology, St. Josef-Hospital, Ruhr-University Bochum, 44791 Bochum, Germany; ^4^St. Elisabeth-Hospital Ruhr-Universität Bochum Große Beckstr, 12,44787 Bochum, Germany

## Abstract

We assess long-term changes in lipid levels in human immunodeficiency disease- (HIV-) infected patients undergoing highly active antiretroviral treatment (HAART) and their association with diabetes mellitus (DM) and thyroid dysfunction. We observed changes in the levels of total cholesterol (TC) and total triglyceride (TG) of 63 HIV-infected patients in the 6 years from starting HAART and analyzed correlations between relevant parameters. TC levels of patients with normal baseline TC levels as well as those diagnosed with DM or impaired fasting glucose (IFG) increased significantly (*P*  < 0.05) as did the TG levels of patients with normal baseline TG levels (*P*  < 0.05). TC levels of patients with hypercholesterolemia in the year HAART was initiated were significantly higher than those of patients with normal baseline TC levels (*P*  < 0.05) for all 6 years. TC levels of patients diagnosed with DM were significantly higher than those with euglycemia (*P*  < 0.05) 2 and 4 years after HAART commencement. Levels of TC, high-density lipoprotein-cholesterol (HDL-C), and low-density lipoprotein-cholesterol (LDL-C) were correlated negatively with viral load, whereas levels of TC and very-low-density lipoprotein-cholesterol (VLDL-C) were correlated positively with CD4+ cell counts before HAART commencement. Linear mixed-effect model demonstrated disturbance of glucose metabolism and HAART containing nevirapine and CD4+ cell count were positively correlated with TC levels after HAART commencement. These findings suggest that there are changes in the lipid levels of patients undergoing HAART, with the potential risk of dyslipidemia.

## 1. Introduction

Acquired immune deficiency syndrome (AIDS) has been considered to be a chronic disease necessitating long-term management rather than an acute fatal disease that requires highly active antiretroviral therapy (HAART). Patients infected with the human immunodeficiency virus (HIV) can survive for long periods, so non-AIDS diseases account primarily for the disease burden of this patient population.

Dyslipidemia is highly prevalent among HIV-infected patients and may contribute to an increased risk of atherogenesis as well as cardiovascular disease (CVD), which is one of the main causes of death in this population [1–3]. Consequently, management of lipid levels is an important aspect of the care of HIV-infected patients.

The characteristics of dyslipidemia associated with HAART include decreased levels of high-density lipoprotein-cholesterol (HDL-C) and increased levels of low-density lipoprotein-cholesterol (LDL-C) and total cholesterol (TC) [4]. HAART can be associated with an increase in insulin resistance [5]. Riddler et al. claimed that significant reductions in serum levels of TC, HDL-C, and LDL-C are seen after HIV infection. However, levels of TC and LDL-C increased notably after HAART initiation but mean levels of HDL-C remained below baseline levels throughout follow-up [6]. Choe et al. evaluated 66 HIV-infected patients and reported that patients diagnosed with hypertriglyceridemia and hypercholesterolemia accounted for 12.3% and 5.8% of cases, respectively [7]. Also, we have shown that serum lipid levels of patients with hyperglycemia are higher than those of patients with euglycemia [8].

Endocrine dysfunction (e.g., thyroid dysfunction) is common in HIV-infected patients [9]. Thyroid hormone has an essential role in lipid metabolism [10]. Several studies have demonstrated that thyroid dysfunction is more common in HIV infected-patients and hypothyroidism is the most common one [11]. Hypothyroidism can lead to increases in TC levels as well as reduction of lipolysis and gluconeogenesis. Patients with severe hyperthyroidism have a higher insulin clearance rate, which gives rise to hyperglycemia [12].

Previously, we undertook cross-sectional studies that did not detail dynamic changes over several years. Consequently, we decided to carry out a longitudinal retrospective study to further observe variations in lipid indices. We hypothesized that the lipid levels of HIV-infected patients undergoing HAART would increase with time and that lipid levels would correlate with glucose metabolism and the levels of thyroid hormones.

## 2. Materials and Methods

### 2.1. Ethical Approval of the Study Protocol

All procedures undertaken in studies involving human participants were in accordance with the ethical standards set by Ruhr University Bochum (Bochum, Germany) and/or national research committees and with the* Declaration of Helsinki*. Written informed consent is not required for this type of study.

### 2.2. Patients

Data for 188 HIV-infected patients undergoing HAART at Saint Josef Hospital, Ruhr University Bochum, from 2000 to 2017, were collected. The final study population was 63 patients.

Patient age, sex, route of transmission, HAART, and the date of diagnosis and the date HAART started were recorded. Levels of TC, triglyceride (TG), glucose, HDL-C, LDL-C, very-low-density lipoprotein-cholesterol (VLDL-C), LDL-C:HDL-C ratio, cluster of differentiation (CD)4+ cell count, and viral load (VL) in the year HAART was started, as well as 2, 4, and 6 years after HAART was started, were collected.

### 2.3. Criteria

Inclusion criteria were patients: (i) diagnosed with HIV infection and undergoing HAART; (ii) aged >18 years; (iii) continuing HAART.

Exclusion criteria were patients (i) with acute HIV infection; (ii) with severe, life-threatening complications; (iii) who were pregnant; (iv) with autoimmune diseases; (v)with data of TC or TG which is incomplete.

### 2.4. Normal Ranges of Lipid Indices Tested

The normal ranges of the indices analyzed were TC, ≤200 mg/dL; TG, ≤150 mg/dL; glucose, 74–106 mg/dL; HDL-C, 33–84 mg/dL; LDL-C, 69–149 mg/dL; LDL-C:HDL-C ratio, 2.5–3.5; thyroid-stimulating hormone (TSH), 0.35–4.94 *μ*IU/mL; free triiodothyronine (FT3), 1.71–3.71 pg/mL; free thyroxine (FT4), 0.70–1.48 ng/dL; CD4+ cells, 300–1400/*μ*L; activated CD8+ human leukocyte antigen D-related (HLA-DR)+ T cells, 300–200 /*μ*L.

### 2.5. Definitions

“Euglycemia” was defined as a glucose level within normal range. “Impaired fasting glucose” (IFG) was denoted as a glucose level of 101–125 mg/dL recorded at least twice. “Diabetes mellitus” (DM) represented a glucose level >125 mg/dL [13]. “Hypothyroidism” referred to a low FT3 level and/or FT4 level with/without a high TSH level. “Hyperthyroidism” was defined as a high FT3 level and/or FT4 level with/without a low TSH level. “Subclinical hypothyroidism” denoted a high TSH level with normal levels of FT3 and FT4. “Subclinical hypothyroidism” represented a low TSH level with normal levels of FT3 and FT4.

### 2.6. Laboratory Tests

VL was determined using a standard reverse transcription-polymerase chain reaction system (COBAS® Amplicor HIV-1 Monitor Test, v1.5; Roche Diagnostic Systems, Branchburg, NJ, USA) with a lower limit of detection of 50 HIV-1 RNA copies/mL. The number of CD4+ cells and activated CD8+ HLA-DR+ T cells was measured by flow cytometry (FACACanto™ II; Becton Dickinson, Franklin Lakes, NJ, USA). Serum levels of TC, TG, HDL-C, LDL-C, and VLDL-C were determined by an automatic biochemical analyzer (AU5800; Beckman Coulter, Brea, CA, USA).

### 2.7. Statistical Analyses

Data analyses were undertaken using SPSS v20 (IBM, Armonk, NY, USA). Numeric variables were expressed as the mean ± standard deviation and qualitative variables as the number of cases (percentages). Repeated-measure tests were used to evaluate trends in lipid levels, after which Student's* t*-test or one-way ANOVA followed by a* post hoc* test was carried out to analyze differences between and among groups. The Pearson correlation coefficient was used to assess the association between data of normal distribution and Spearman correlation coefficient to nondistribution data. Linear mixed-effects model considering sex, diagnosis of glycometabolism, age, taking nevirapine, or not as fixed effects, and lgVL and CD4+ cell counts as random effects was made to assess the correlation between some essential data and TC levels. P < 0.05 (two-tailed) was considered significant.

## 3. Results

### 3.1. Clinical Information

Table 1 showed basic clinical information of patients before HAART commencement. Of the 63 patients enrolled, 82.5% were male. The mean age of the study cohort was 40.47 ± 9.40 years. Forty-two patients were infected with HIV after male homosexual sex, 16 after heterosexual sex, and five by an unknown route. Thirty-five patients received two nucleoside reverse transcriptase inhibitor (NRTIs) plus one nonnucleoside reverse transcriptase inhibitor (NNRTIs). Duration of diagnosis before HAART commencement is 2.43 ± 3.79 years. The baseline TC levels of 50 patients were normal and 13 were higher than the upper limit of normal. Based on the levels of thyroid hormones, 11 patients were diagnosed as having hypothyroidism, five with subclinical hypothyroidism, and two with hyperthyroidism. According to glucose levels, 14 were diagnosed with DM and 34 with IFG (Table 1). The VL of 45 patients was below the limit of detection 2 years after HAART commencement.

### 3.2. Changes in TC Levels

Patients were divided into two groups according to their baseline TC level. Fifty patients had a baseline TC level within normal range (normal group) and 13 had a baseline TC level higher than the upper limit of normal (hypercholesterolemia group).

**Figure 1:**
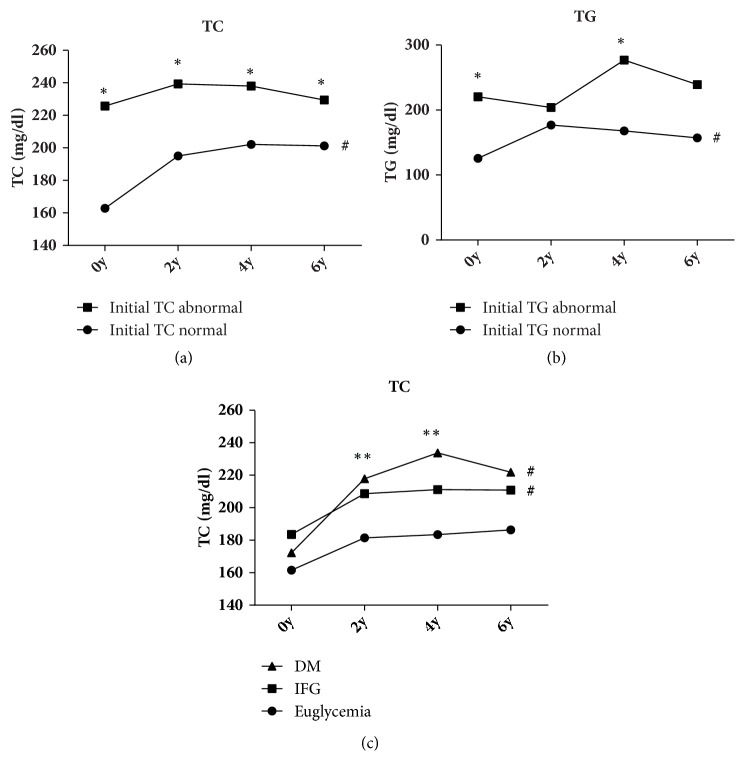
**Changes in levels of TC and TG over 6 years within different groups**. Legends: TC: total cholesterol; TG: total triglyceride; DM: diabetes mellitus; IFG: impaired fasting glucose. ^∗^P < 0.05, initial TC/TG abnormal* vs*. initial TC/TG normal; ^∗∗^P < 0.05, DM* vs*. euglycemia; ^#^P < 0.05, 2, 4, and 6 years* vs*. the year HAART started.

There were significant changes in the TC levels of the two groups with increasing time (P < 0.05) and the TC levels of the hypercholesterolemia group were significantly higher than those of the normal group (P < 0.05) over the whole 6 years (ESM Table 1a). In addition, the TC level (in mg/dL) of the hypercholesterolemia group was significantly higher than that of the normal group for each year (225.77 ± 27.59* vs.* 162.84 ± 36.15; 239.38 ± 43.38* vs*. 195.06 ± 45.29; 238.00 ± 33.09* vs*. 202.18 ± 50.38; 229.46 ± 37.81* vs*. 201.22 ± 41.08; P < 0.05) (ESM Table 1a, Figure 1(a)). For the normal group, changes in TC levels over the 6 years were significant (P < 0.05) and TC levels 2, 4 and 6 years after HAART commencement were remarkably higher than that of the year in which HAART was started. Over the same period, the TC levels of the hypercholesterolemia group did not exhibit a significant difference (P > 0.05).

Similarly, we grouped patients according to their glucose metabolism. The TC levels of patients with different glucose metabolism changed significantly over time (P < 0.05) (ESM Table 2, Figure 1(c)) and there were significant differences among the DM, IFG, and euglycemia groups (P < 0.05). The TC levels (in mg/dL) of the DM group were significantly higher than those of the euglycemia group 2 and 4 years after HAART commencement (217.86 ± 35.36* vs*. 181.47 ± 36.01, P = 0.032; 233.79 ± 31.45* vs*. 183.47 ± 55.56, P < 0.05). The TC levels in the euglycemia group increased over years without a significant change (P > 0.05). However, the TC levels of the IFG and DM groups 2, 4, and 6 years after HAART commencement were significantly higher than those in the year HAART was started (P < 0.05). Also, the TC levels of IFG and DM groups increased significantly from the year HAART started to the fourth year (P < 0.05) and decreased in the sixth year, but nonsignificantly (P > 0.05).

TC levels did not show a significant difference in groups with different thyroid function (P > 0.05).

### 3.3. Changes in TG Levels

Patients were divided into two groups according to their baseline TG level. Forty-one patients had a normal baseline level (normal group) and 22 had a TG level higher than the upper limit of normal (hypertriglyceridemia group).

The TG levels of the hyperglyceridemia group were significantly higher than those of the normal group (P < 0.05). The TG levels (in mg/dL) of the hyperglyceridemia group were higher than that of the normal group for each year since HAART commencement (220.27 ± 86.68* vs.* 125.73 ± 91.32, P < 0.05; 203.95 ± 159.59* vs.* 176.80 ± 158.32, P > 0.05; 276.91 ± 192.21* vs* 167.76 ± 170.96, P < 0.05; 239.05 ± 203.49* vs* 157.10 ± 144.10, P > 0.05) (ESM Table 1b, Figure 1(b)). The TG levels of the normal group changed significantly over the 6 years of HAART (P < 0.05).

There were no significant differences among patients with different glucose metabolism and thyroid function (P > 0.05).

### 3.4. Changes in CD4+ Cell Counts

**Figure 2:**
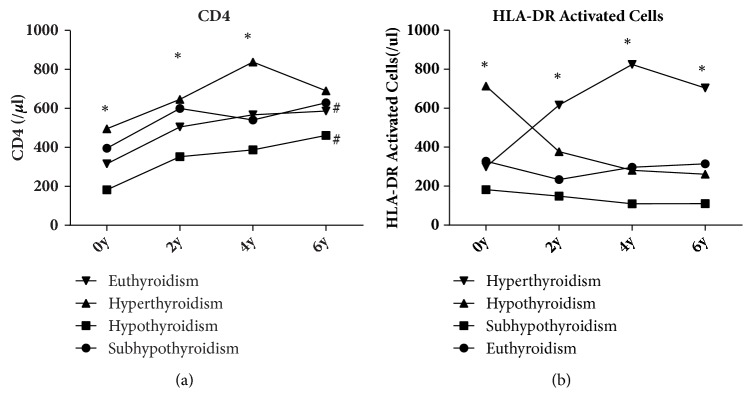
**Variations in CD4+ cell counts and activated CD8 + HLA-DR T-cell counts over 6 years for patients with different thyroid function**. Legends: HLA-DR activated cells: activated CD8+ human leukocyte antigen D-related T cells. ^∗^P < 0.05, hyperthyroidism* vs*. hypothyroidism* vs*. sub-hypothyroidism* vs*. euthyroidism; ^#^P < 0.05, 2, 4, and 6 years* vs*. the year HAART started.** Detailed description:** (a): Picture (a) demonstrates that the CD4+ cell counts of patients diagnosed with hypothyroidism were significantly lower (P < 0.05) than those of patients in the other groups in the year HAART started, significantly lower (P < 0.05) than those diagnosed with euthyroidism and subclinical hypothyroidism in 2 years after HAART commencement, and significantly lower (P < 0.05) than those diagnosed with euthyroidism and hyperthyroidism 4 years after HAART commencement. (b): Picture (b) demonstrates that the activated CD8 + HLA-DR T-cell counts of patients diagnosed with hypothyroidism were significantly higher (P < 0.05) than those diagnosed with euthyroidism or subclinical hypothyroidism in the year HAART started. Since year 2, the activated CD8 + HLA-DR T-cell counts of patients diagnosed with hyperthyroidism were significantly higher than those of the other groups (P < 0.05).

Changes in CD4+ cell counts and activated CD8+ HLA-DR T-cell counts were analyzed to assess therapeutic effects and patient immunity (ESM Table 3, Figure 2).

CD4+ cell counts (per *μ*L) increased significantly over the years (307 ± 185.56* vs*. 489.94 ± 200.24* vs*. 542.13 ± 215.85* vs*. 571.21 ± 230.96, P < 0.05). CD4+ cell counts were compared among patients with different thyroid function. There was a significant difference among groups with different thyroid function until 4 years after HAART commencement (P < 0.05) and this significance disappeared at 6 years after HAART commencement. The hypothyroidism group had the lowest CD4+ cell count (per *μ*L) over the whole 6 years, and it was significantly lower than that of the euthyroidism group in the year HAART started as well as 2 and 4 years after (181.91 ± 135.79* vs.* 315.49 ± 177.97, 352.27 ± 142.25* vs.* 504.47 ± 199.76, 387.00 ± 170.44* vs. *567.11 ± 205.39, P < 0.05; 461.09 ± 203.43* vs.* 586.42 ± 223.93, P > 0.05). However, there were no significant differences in groups with different glucose metabolism. The activated CD8 + HLA-DR T-cell counts of patients diagnosed with hypothyroidism were significantly higher (P < 0.05) than those of patients diagnosed with euthyroidism or subclinical hypothyroidism in the year HAART started. From 2 years since HAART commencement, the activated CD8 + HLA-DR T-cell counts of patients diagnosed with hyperthyroidism were significantly higher than those of patients diagnosed with hypothyroidism, euthyroidism, or subclinical hypothyroidism (P < 0.05).

### 3.5. Correlations between Indices before HAART

Correlations between indices before patients started HAART were done to analyze relationship of HIV infection and TC levels, as HAART may change the pattern of patients' lipid metabolism and VL levels. Levels of TC, HDL-C, and LDL-C correlated negatively with VL (R_1_ = −0.511, R_2_ = −0.516, R_3_ = −0.396, and P < 0.05) (Table 2). Levels of TC and VLDL-C correlated positively with the CD4+ cell count (R_1_ = 0.499, R_2_ = 0.621, and P < 0.05). The CD4+ cell count correlated negatively with VL (R = −0.512, P < 0.05). In addition, levels of FT3 and VLDL-C were correlated positively with CD4+ cell count (R_1_ = 0.596, R_2_ = 0.621, and P < 0.05).

### 3.6. Factors Associated with TC

The result of linear mixed-effect model was made to explore factors influence TC levels in the six years since patients received HAART (Table 3). The result showed that patients diagnosed as IFG and DM compared with euglycemia ( *β*1 = 27.84, *β*2 = 45.41, and P < 0.05), HAART including nevirapine (*β* = 24.79, P < 0.05), and CD4+ cell count (*β* = 0.004, P < 0.05) positively correlated with TC levels in the six years since HAART commencement.

## 4. Discussion

In general, in the present study, the VL of most patients was below the limit of detection and CD4+ cell counts increased continually after treatment. These observations suggest that a favorable clinical curative effect had been elicited.

Changes in lipid metabolism after HAART were observed in our study. However, the data for the levels of HDL-C, LDL-C, VLDL-C, and LDL-C:HDL-C ratio were incomplete. Hence, changes in the levels of TC and TG over time were analyzed. We found that several patients with baseline levels of TC and TG within normal ranges could suffer hypercholesterolemia or hypertriglyceridemia over 6 years of HAART. The levels of TC and TG of these patients increased remarkably overall. We assumed that physicians monitored the levels of TC and TG upon acceptance of HAART irrespective of whether patients had hypercholesterolemia or hypertriglyceridemia at that time.

The TC levels of patients with hyperglycemia (IFG and DM) also increased significantly in the first 4 years from HAART commencement. The interactions between disturbances in glucose metabolism and lipid metabolism are complicated. One mainstream hypothesis is that insulin resistance can lead to dyslipidemia [14]. We found no significant relationship between levels of TC and glucose before HAART commencement but noted a significant difference among patients with different glucose metabolism as well as positive correlation between pathoglycemia (IFG and DM) with TC levels, both implying that a disturbance in glucose metabolism (and not the glucose level) affected TC levels. Thus, we suggest that the insulin resistance of patients with IFG or DM results in an increase in TC levels. Hyperglycemia and hyperlipidemia are risk factors for CVD. Hence, patients with IFG or DM should avoid CVD risk factors as much as possible and physicians should take this into consideration when proposing the HAART regimen. Besides, it is recognized that protease inhibitors are responsible for increases in levels of TC and TG [15].

We observed that the LDL level was correlated positively with the TSH level before HAART commencement, although there were no remarkable differences among patients with different thyroid function. According to Yingyun Gong and his colleagues, TSH elevated concentration of LDL-c by upregulating hepatic PCSK9 expression [16]. However, Goldberg et al. demonstrated that T3 treatment leads to a remarkable reduction in TC levels [17]. Yazbeck and colleagues stated that the TC level of patients with hypothyroidism increased and that treatment with thyroid hormone would help to lower the TC level; they attributed this phenomenon to the higher morbidity of atherosclerosis in patients with hypothyroidism [18]. Al-Tonsi et al. demonstrated that the levels of TC and TG of patients with hypothyroidism increased and that the TC level was correlated significantly with levels of T4 and TSH [19]. Taken together, these data suggest that patients with thyroid dysfunction are more susceptible to dyslipidemia.

We noted significant changes in levels of TC and TG from the year HAART started to 2 years after HAART commencement in patients with normal baseline levels of TC and TG, as well as those diagnosed with IFG or DM. However, there were no significant changes from 2 years of HAART commencement, such as between year 2 and year 4. We suggest that disorders in lipid metabolism occur mainly in the early years after HAART commencement.

Levels of TC, HDL-C, and LDL-C were correlated negatively with VL before HAART commencement in the present study. HIV seroconversion was ascribed to reductions in levels of TC, HDL-C and LDL-C according to Multicenter AIDS Cohort Study [6]. HDL-C facilitates foam cells to excrete cholesterol, has anti-inflammatory and anti-thrombotic effects, protects endothelial cells, and prevents CVD. Hence, eliminating a virus is crucial for CVD prophylaxis. In the present study, TC levels were correlated negatively with VL, which was in accordance with the work of Baker and colleagues [20]. Madeddu and coworkers stated that HIV-infected patients not undergoing HAART suffer hypocholesterolemia [9]. TC levels were correlated negatively with the HIV load before HAART commencement, which could influence the cholesterol excretion of monocytes by downregulating expression of key cholesterol efflux transporter genes such as ABCA1 and ABCG1 in monocytes and affect expression of *β*-Hydroxy *β*-methylglutaryl-CoA (the rate-determining enzyme responsible for cholesterol biosynthesis). Suppression of cholesterol excretion reduces TC levels in plasma and promotes the formation of foam cells, which accelerates atherosclerosis development [21]. CD4+ cell count was positively correlated with TC and VLDL levels before HAART commencement. Furthermore, during six years since HAART started, there was still a positive correlation between CD4+ cell count and TC levels. This was in accordance with the findings of Melaku Adal et al [22].

Long-term HAART might result in dyslipidemia. Nevirapine was found to be positively correlated with TC levels in this study while there was no statistical significance between tenofovir and emtricitabin with TC levels. However, Alex Marzel et al. concluded that HAART containing tenofovir was negatively correlated with TC levels [23]. Fasting levels of triglycerides, TC, and LDL-C of patients taking stavudine have been reported to be higher than those for patients taking tenofovir [24].

The main strength of our study was that it was a 6-year follow-up study with complete repeat measurement of the levels of TC and TG as well as HIV viral load. Moreover, with continuous and good compliance, therapeutic effect was satisfactory.

There were three main limitations in our study. First, we found the TC level 6 years after HAART commencement to be lower than that at 4 years, but it increased persistently in the first 4 years from HAART commencement. This phenomenon may have been because the TC level benefited from HIV clearance, an increase in the CD4+ cell count with improvement of disease, and supplementation with thyroid hormones, or it was temporary, in which case the TC level could increase again. Therefore, following up TC levels for a longer time would be a good strategy. Second, the number of patients diagnosed with subclinical hypothyroidism or hyperthyroidism was few. As a result, enlarging the sample size is crucial to increase the reliability of our data. Finally, levels of TSH, FT3, FT4, HDL-C, LDL-C, VLDL-C, and the LDL-C:HDL-C ratio were not detected regularly, so we could not observe changes in these indices.

## 5. Conclusions

HIV-infected patients tend to suffer from dyslipidemia, especially those with hyperglycemia and thyroid dysfunction. It may be necessary to prophylactically treat this population with antilipidemic medicine as even patients with normal TC are inclined to dyslipidemia.

## Figures and Tables

**Table 1:** Clinical information for 63 patients before HAART commencement.

Variables	Mean (±SD) or number (%)

Age (years)	40.47 ± 9.40
Sex	
Male	52 (82.5%)
Female	11 (17.5%)
HIV transmission category	
Male homosexual	42 (66.7%)
Heterosexual	16 (25.4%)
Unknown	5 (7.9%)
HAART regimen	
2 NRTI + PI	14 (22.2%)
2 NRTI + NNRTI	35 (55.6%)
2 NRTI + II	3 (4.8%)
Other	11 (17.5%)
Duration of diagnosis (years)	2.43 ± 3.79
Baseline TC level	
Normal	50 (79.4%)
Increased	13 (20.6%)
Baseline TG level	
Normal	41 (65.1%)
Increased	22 (34.9%)
Thyroid function	
Normal	45 (71.4%)
Abnormal	18 (28.6%)
Sub-hypothyroidism	5 (7.9%)
Overt hypothyroidism	11 (17.5%)
Overt hyperthyroidism	2 (3.2%)
Glucose metabolism	
Euglycemia	15 (23.8%)
IFG	34 (54.0%)
DM	14 (22.2%)
HBV or HCV co-infection	
neither	48 (76.2%)
HBV	13 (20.6%)
HCV	2 (3.2%)

HIV: human inmunodeficiency virus; HAART: highly active antiretroviral therapy; NRTI: nucleoside reverse transcriptase inhibitor; PI: protease inhibitor; NNRTI: non-nucleoside reverse transcriptase inhibitor; II: integrase inhibitor; TC: total cholesterol; TG: total triglyceride; IFG: impaired fasting glucose; DM: diabetes mellitus; HBV: hepatitis-B virus; HCV: hepatitis-C virus.

**Table 2:** Correlation between relevant parameters before HAART.

	r	P

TC & TG	0.339	0.015
TC & HDL-C	0.456	0.019
TC & LDL-C	0.856	0.000
TC & lgVL	-0.511	0.000
TC & CD4	0.499	0.000
TG & VLDL-C	0.693	0.000
HDL-C & lgVL	-0.516	0.007
VLDL-C & CD4	0.621	0.001
LDL-C & TSH	0.555	0.021
LDL-C & lgVL	-0.396	0.045
FT3 & CD4	0.596	0.002
LgVL & CD4	-0.512	0.000

HAART: highly active antiretroviral treatment; TC: total cholesterol; TG: total triglyceride; HDL-C: high-density lipoprotein-cholesterol; LDL-C: low-density lipoprotein-cholesterol; VL: viral load; VLDL-C: very-low-density lipoprotein-cholesterol; TSH: thyroid-stimulating hormone; FT3: free triiodothyronine.

**Table 3:** Model-based estimated factors correlated with TC levels.

	estimate	Standard error	P value

Sex ( male vs. female)	4.44	14.17	0.755
IFG vs. euglycemia	27.84	11.09	0.014
DM vs. euglycemia	45.41	11.80	0.000
age	0.45	0.49	0.355
nevirapine	24.79	11.07	0.028
lgVL	39.10	22.12	0.077
CD4	0.004	0.001	0.000

TC: total cholesterol; IFG: impaired fasting glucose; DM: diabetes mellitus; VL: viral load.

## Data Availability

The SPSS data used to support the findings of this study are available from the corresponding author upon request.

## Supplementary Materials

Supplementary Materials See Table 1a-3 in the Supplementary Material for comprehensive image analysis.
