# Supplementary material for: Changes in Lipid Indices in HIV+ Cases on HAART
Source: Biomed Res Int. 2019 Feb 5;2019:2870647. doi: 10.1155/2019/2870647 (PMC6379880; doi:10.1155/2019/2870647)
Supplement: Supplementary Materials — See Table 1a-3 in the Supplementary Material for comprehensive image analysis. [file 2870647.f1.doc]

Table 1a. Baseline TC levels at different times

|  | Initial TC normal  (n = 50) (mg/dL) | Initial TC abnormal (n = 13) (mg/dL) | Total  (n = 63) (mg/dL) | P |
| --- | --- | --- | --- | --- |
| TC at 0 years | 162.84±36.15 | 225.77±27.59 | 175.83±42.88 | 0.000 |
| TC at 2 years | 195.06±45.29 | 239.38±43.38 | 204.21±48.11 | 0.002 |
| TC at 4 years | 202.18±50.38 | 238.00±33.09 | 209.57±49.31 | 0.018 |
| TC at 6 years | 201.22±41.08 | 229.46±37.81 | 207.05±41.75 | 0.029 |
| P | 0.000 | 0.333 | 0.000 |  |

Table 1b. Baseline TG levels at different times

|  | Initial TG normal  (n = 41) (mg/dL) | Initial TG abnormal (n = 22) (mg/dL) | Total  (n = 63) (mg/dL) | P |
| --- | --- | --- | --- | --- |
| TG at 0 years | 125.73±91.32 | 220.27±86.68 | 158.76±99.95 | 0.000 |
| TG at 2 years | 176.80±158.32 | 203.95±159.59 | 186.29±158.01 | 0.520 |
| TG at 4 years | 167.76±170.96 | 276.91±192.21 | 205.87±184.72 | 0.024 |
| TG at 6 years | 157.10±144.10 | 239.05±203.49 | 185.71±170.21 | 0.068 |
| P | 0.003 | 0.170 | 0.018 |  |

Table 2 TC levels for different glucose metabolism

|  | Euglycemia  (n = 15) (mg/dL) | IFG  (n = 34) (mg/dL) | DM  (n = 14) (mg/dL) | Total  (n = 63) (mg/dL) | P |
| --- | --- | --- | --- | --- | --- |
| TC at 0 years | 161.53±34.90 | 183.58±45.51 | 172.29±42.37 | 175.83±42.88 | 0.241 |
| TC at 2 years | 181.47±36.01 | 208.62±54.51 | 217.86±35.36 | 204.21±48.11 | 0.027(0, 2) |
| TC at 4 years | 183.47±55.56 | 211.12±48.04 | 233.79±31.45 | 209.57±49.31 | 0.020(0, 2) |
| TC at 6 years | 186.40±31.01 | 210.86±44.04 | 221.86±40.05 | 207.05±41.75 | 0.058 |
| P | 0.069 | 0.000 | 0.000 | 0.033 |  |

Table 3. CD4+ counts and activated HLA-DR T-cell counts for different thyroid function

|  | Euthyroidism (n = 45) (mg/dL) | Sub-  hypothyroidism (n = 5) (mg/dL) | Hypothyroidism (n = 11) (mg/dL) | Hyperthyroidism(n = 2) (mg/dL) | P |
| --- | --- | --- | --- | --- | --- |
| **CD4** |  |  |  |  |  |
| 0 years | 315.49±177.97 | 395.20±198.00 | 181.91±135.79 | 495.50±275.06 | 0.034 (0, 2; 1, 2; 2, 3) |
| 2 years | 504.47±199.76 | 599.60±217.59 | 352.27±142.25 | 646.00±107.48 | 0.039 |
| 4 years | 567.11±205.39 | 540.20±261.72 | 387.00±170.44 | 838.00±8.49 | 0.015 |
| 6 years | 586.42±223.93 | 628.80±348.68 | 461.09±203.43 | 690.50±108.19 | 0.321 |
| P | 0.000 | 0.330 | 0.000 | 0.380 |  |
| **HLA-DR** |  |  |  |  |  |
| 0 years | 327.60±326.31 | 182.25±101.79 | 713.64±421.62 | 298.50±10.61 | 0.009 |
| 2 years | 233.89±211.54 | 148.75±125.27 | 377.09±261.97 | 616.00±217.79 | 0.024 |
| 4 years | 297.02±255.26 | 109.50±59.22 | 281.00±247.89 | 824.50±236.88 | 0.012 |
| 6 years | 314.24±198.77 | 110.50±49.60 | 261.64±170.30 | 703.50±335.88 | 0.006 |
